# The *e*-Index, Complementing the *h*-Index for Excess Citations

**DOI:** 10.1371/journal.pone.0005429

**Published:** 2009-05-05

**Authors:** Chun-Ting Zhang

**Affiliations:** Department of Physics, Tianjin University, Tianjin, China; Université de Toulouse, France

## Abstract

**Background:**

The *h*-index has already been used by major citation databases to evaluate the academic performance of individual scientists. Although effective and simple, the *h*-index suffers from some drawbacks that limit its use in accurately and fairly comparing the scientific output of different researchers. These drawbacks include information loss and low resolution: the former refers to the fact that in addition to *h* ^2^ citations for papers in the *h*-core, excess citations are completely ignored, whereas the latter means that it is common for a group of researchers to have an identical *h*-index.

**Methodology/Principal Findings:**

To solve these problems, I here propose the *e*-index, where *e* ^2^ represents the ignored excess citations, in addition to the *h* ^2^ citations for *h*-core papers. Citation information can be completely depicted by using the *h*-index together with the *e*-index, which are independent of each other. Some other *h*-type indices, such as *a* and *R*, are *h*-dependent, have information redundancy with *h*, and therefore, when used together with *h*, mask the real differences in excess citations of different researchers.

**Conclusions/Significance:**

Although simple, the *e*-index is a necessary *h*-index complement, especially for evaluating highly cited scientists or for precisely comparing the scientific output of a group of scientists having an identical *h*-index.

## Introduction

The *h*-index, proposed by Hirsch [1], [2], has already been used by major citation databases, such as Web of Science and Scopus, to evaluate the academic performance of individual scientists. Although effective and simple, the *h*-index suffers from some drawbacks that limit its use in accurately and fairly comparing the scientific output of different researchers. Many of these drawbacks have been pointed out in literatures, and consequently some *h*-type indices were proposed to overcome these drawbacks [3]–[14]. For reviews, refer to [15]–[17].

Here I emphasize that two disadvantages of the *h*-index have not yet been sufficiently overcome. The first disadvantage is the loss of citation information, i.e., in addition to the *h* ^2^ citations that can be inferred from the *h*-index, excess citations are completely ignored. Due to this drawback, comparisons based on *h*-index alone can be misleading, because researchers having a lower *h*-index can in fact have much more citations than those having a higher *h*-index.

The second drawback of the *h*-index is the low resolution, resulting from its low potential. The *h*-index, composed of natural numbers, has a much lower potential than a set of real numbers. Furthermore, the *h*-index has a relatively narrow range. For instance, Dr. Edward Witten had the highest *h*-index, 110, among physicists all over the world [1]. In fact, in any field, scientists having an *h*-index larger than 100 (at least 10,000 citations) are rare. Therefore, due to low resolution, it is quite common for a group of scientists to have an identical *h*-index. This paper is devoted to solving the two aforementioned problems by introducing the *e*-index, a real number, to complement the *h*-index for the ignored excess citations.

## Results and Discussion

### Definitions of the *e*-index

In what follows, we study only the citations received by papers in the *h*-core, all of a researcher's papers having at least *h* citations [18]. Using the *h*-index, the only citation information that can be inferred is 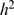, i.e., at least *h* ^2^ citations have been received, and additional citations for papers in the *h*-core are completely ignored. Here we define the *e*-index to complement the *h*-index for the ignored excess citations. The excess citations received by all papers in the *h*-core, denoted by 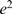, are
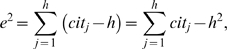
(1)where 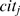 are the citations received by the *j* ^th^ paper and 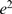 denotes the excess citations within the *h*-core. Letting
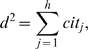
(2)we have
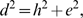
(3)or
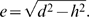
(4)Note that 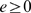, and 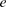 is a real number. Accordingly,
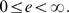
(5)


### A geometrical explanation of the *e*-index

**Figure 1:**
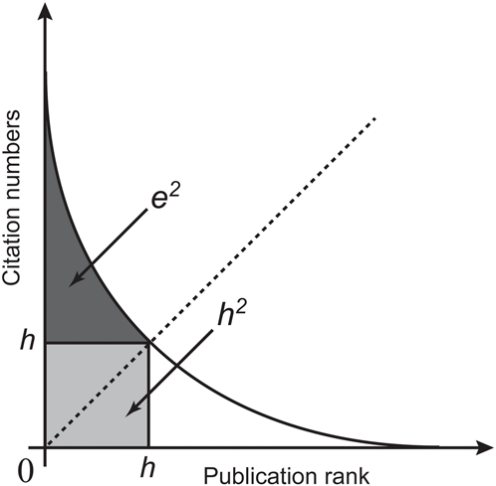
A geometrical explanation of the *e*-index. Without losing generality, we assume that 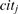, 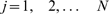, can be represented by a smooth function 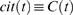, 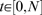. A typical 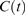 curve is shown here, where 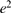 is equal to the area of the dark gray region.

Without losing generality, we assume that 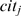, 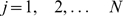, can be represented by a smooth function 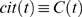, 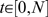, where 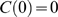, and 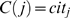, 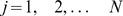. Based on the function 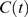, we will give a geometrical explanation about the above formulas.
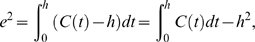
(6)i.e., 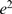 is equal to the area of the dark gray region in Figure 1.

I emphasize that 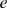 is independent of *h*, and 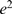 represents the net excess citations received by all papers in the *h*-core, in addition to *h* ^2^ citations. Note that the larger the 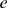, the larger the net excess citations, and hence more severe of the loss of citation information when using the *h*-index alone. In other words, when the *h*-index is used to evaluate individual scientists, the smaller the *e*, the more reliable the *h*-index is. In an extreme case, when 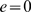, which is highly unlikely in reality, the *h*-index completely describes the citation information for papers in the *h*-core. Otherwise, when 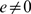, the *h*-index always losses citation information, which is complemented by the *e*-index.

### Numerical relations between the *e*-index and some other *h*-type indices

**Figure 2:**
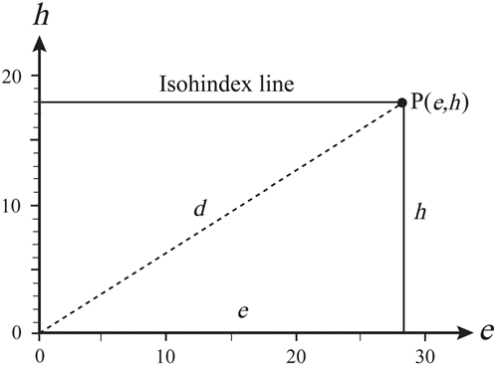
A Descartes coordinate system for the *h*-*e* plane. The x-axis indicates the *e*-index, and the *y*-axis indicates the *h*-index. All the horizontal lines are called isohindex lines, on which all the mapping points have identical *h*-index. One of the isohindex lines is shown. The *R*-index is equal to 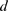, the Euclidean distance between the point 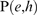 and the origin.

The relations between the *e*-index and some other *h*-type indices, including the *a*-index [7] and the *R*-index [14], are presented briefly as follows. A plane is spanned by *h* and *e*, called the 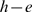 plane (Figure 2). A point 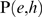 in the 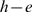 plane represents the overall information of citations received by all papers in the *h*-core. It is interesting to point out that the Euclidean distance between the origin and the point 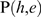 is equal to
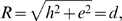
(7)where the *R*-index here is given a geometrical meaning (Figure 2).

From eq. (3), it is found
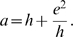
(8)


The 4 indices being discussed, *h*, *e*, *a* and *R*, can be divided into two types, fundamental ones and derived ones. A fundamental index satisfies following conditions (i) it is an independent variable (ii) it can be used to derive other indices. Here *h* and *e* are fundamental indices, because they are independent of each other, and they can be used to derive *a* and *R*. In contrast, *a* and *R* are derived indices, because they are dependent on *h* and *e*, which are not derivable given either *a* or *R*.

Let
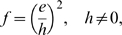
(9)where *f* denotes the fold of excess citations over the *h* ^2^ citations received for papers in the *h*-core. The total citations received in the *h*-core are equal to 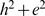, as shown in Figure 1. Therefore, the combination (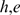) provides complete citation information in the *h*-core. In contrast, *a* and *R* are derived indices, and they therefore have information redundancy with *h*. When *a* or *R* is used together with *h*, the information redundancy masks the *f* values, i.e., the real fold of excess citations over the *h* ^2^ citations are less than the real ones, which will be exemplified by comparisons of citations for some scientists in the following section.

### Comparison of the academic performance of scientists within an isohindex group

The mapping points P(*e*, *h*) can only be situated on the horizontal lines in the *h*-*e* plane with *h* = 1, 2, …, H, where H is the largest value of the *h*-index, given a group of scientists. All of the points on the same horizontal line have an identical *h*-index. For convenience, this horizontal line is called an isohindex line, where “isohindex” denotes an identical *h*-index. One of such isohindex lines is shown in Figure 2. We further define the isohindex group as follow: A group of scientists having an identical *h*-index is said to be within an isohindex group. To compare the academic performance of scientists belonging to the same isohindex group, the *h*-index is inadequate, and the *e*-index becomes especially necessary.

The journal *Chemistry World* published a list of chemists with high *h*-indices [19]. As an example, we chose from the list two chemists both having an *h*-index of 51 (Table 1). Although having an identical *h*-index, the second researcher in fact had much more citations than the first researcher. The *e*-indices for the second and first researchers are 54.73 and 31.10, respectively, and (54.73/31.10)^2^ = 3.1, indicating that the citations ignored by the *h*-index for the second researcher are more than 3 times of those of the first researcher.

**Table 1:** The *e*-index and some derived *h*-type indices for three famous chemists.^a^

No.	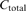	*h*	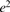	*f*	*e*	*a*	*R*
1	3568	51	967	0.37	31.10	69.96	59.73
2	5596	51	2995	1.15	54.73	109.73	74.81
3	15496	50	12996	5.20	114.00	309.92	124.48

a Note that 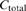 is the total citations received by all papers in the *h*-core, and 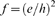, 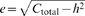, 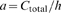 and 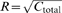.

The merit of using the *e*-index is that 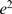 is strictly equal to the net excess citations received for all the papers in the *h*-core, whereas *a* and *R* are not. Both *a* and *R* are derived indices, and they all include contribution from both *h* ^2^ citations and the net excess citations (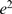), and they are dependent on *h* and *e*, while *e* is independent of *h*. Consequently, using *a* or *R* together with the *h*-index to evaluate the performance of scientists within an isohindex group can lead to unrealistic result.

Compared with the first researcher, the second one had a more than 2-fold increase in net excess citations, however, *a* and *R* only increased by 0.57- and 0.25-fold, respectively. Therefore, *a* and *R* indices mask the real difference in ignored excess citations, and the *e*-index is more objective and precise, when used together with the *h*-index, in comparing the citation information for researchers within an isohindex group.

The third researcher listed in Table 1 is the famous chemist, Dr. Berni Alder, who pioneered computer simulation. It is noteworthy that the *h*-index severely underestimates the scientific impact of him. Although having an *h*-index of 50, Dr. Alder's total citations were much more than many researchers having an *h*-index of 50 or even more than 50. For instance, the total citations of Dr. Alder were more than 4 times of those for the first researcher, who had a higher *h*-index, 51. The *e*-index for Dr. Alder was 114.0 and *f* = 5.2, indicating that the ignored excess citations by the *h*-index were more than 5 times of the *h* ^2^ citations, highlighting the need for using the *e*-index.

### Loss of citation information by the *g*-index

The *e*-index proposed here is aimed at considering the contributions of excess citations, which are mainly from highly cited papers. It is necessary to mention the *g*-index, which was proposed as being “sensitive to the level of the highly cited papers” [5]. The *g*-index is defined as “the highest number of g of papers that together received g^2^ or more citations” [5]. Although having some advantages, the *g*-index also suffers from the loss of citation information in many important cases, especially for distinguished scientists (most of whose papers are highly cited). For instance, for any 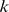, if
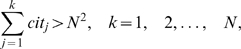
(10)then the *g*-index has no definition. In fact, for any *N* conditions in eq. (10), the *g*-index can have no definition. Among the *N* conditions in eq. (10), the strongest condition is
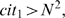
(11)and the weakest condition is
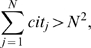
(12)Eq.s (10), (11) and (12) are associated with many important cases. For example, Dr. Frederick Sanger is an outstanding scientist, who won the Nobel Prize twice. He has published 30 papers (N = 30), and the citation number for one of his paper is 63781, much more than 30^2^ = 900, indicating that the condition in eq. (11) is satisfied.

Noticing this problem, Egghe later proposed two options [20]: “we can define g = T [T denotes the total number of papers], or better […], we can add […] fictitious articles with zero citations: We add enough of these “articles” so that […] we denote by T the new number of articles (including the fictitious ones)”.

By the option 1 the *g*-index for Dr. Sanger is 30, where total citations 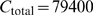, and therefore, about 99% of citations are ignored by the *g*-index (30^2^/79400). Therefore the option 1 could lead to the loss of citation information, especially for distinguished scientists; and in such cases, the more highly cited, the more of the loss of information. By the option 2, the *g*-index is always equal to [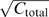], where [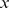] is the integer part of 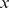. Therefore, for Dr. Sanger, *g* = 281, suggesting that about 90% of papers (1–30/281) are fictitious. If the option 2 is adopted, by the *g*-index alone, there is no way for users to know, for a scientist being evaluated, how many papers are real, and how many papers are fictitious; this will confuse users, as an old saying goes “Fiction in fact, then fact becomes fiction”. Therefore, both options seem not ideal. Here I suggest that the use of an *e*-like index to denote the loss of citations would be another way to solve the above problem of the *g*-index.

### A simple mathematical model

Based on the citation curve 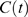, the *h* and *e*-indices can be calculated. Here we study only a simple mathematical model. We assume that
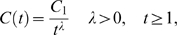
(13)where 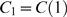 is the maximum citations received by a paper in the *h*-core. First of all, we assume 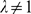. According to the definition of the *h*-index, we have 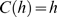, leading to the result
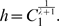
(14)


Based on eq. (6), we find
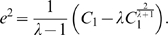
(15)


When 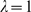, similarly we have
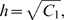
(16)

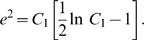
(17)


The parameters could be estimated from eq. (14), and it was found that 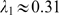, 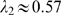, and 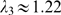, respectively, for the 3 chemists listed in Table 1. I emphasize that when 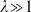, 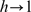, and then the *h*-index becomes unreliable in reflecting the academic performance. For example, letting 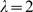, and assuming 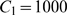, we find 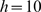, and 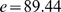. Consequently, 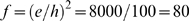. This result shows that even when 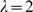, the ignored excess citations (80000) are much more than the *h* ^2^ citations (100).

### Concluding remarks

The *h*-index has already been used by major citation databases to evaluate the academic performance of individual scientists. Because of the loss of citation information, comparisons based on the *h*-index alone can be misleading, as exemplified by Dr. Alder, whose total citations are much more than those of many researchers having higher *h*-indices; the ignored excess citations (*e* ^2^) are more than 5 times of *h* ^2^ citations. Therefore, for accurate and fair comparisons, it is necessary to use the *e*-index together with the *h*-index. Some other *h*-type indices, such as *a* and *R*, are *h*-dependent, have information redundancy with *h*, and therefore, when used together with *h*, mask the real differences in excess citations of different researchers. Although simple, the *e*-index is a necessary *h*-index complement, especially for evaluating highly cited scientists or for precisely comparing the scientific output of a group of scientists having an identical *h*-index.

## Materials and Methods

The 3 scientists listed in Table 1 were from the *h*-index ranking published by Chemistry World [19]. The third scientist in Table 1 was Dr. Berni Alder. The citations for those listed in Table 1 and for Dr. Frederick Sanger were obtained from Web of Science in March, 2009.

## References

[1] Hirsch JE (2005). An index to quantify an individual's scientific research output.. Proceedings of the National Academy of Sciences of the United States of America.

[2] Hirsch JE (2007). Does the h index have predictive power?. Proceedings of the National Academy of Sciences of the United States of America.

[3] Kelly CD, Jennions MD (2006). The h index and career assessment by numbers.. Trends in Ecology & Evolution.

[4] Burrell QL (2007). Hirsch index or Hirsch rate? Some thoughts arising from Liang's data.. Scientometrics.

[5] Egghe L (2006). An improvement of the h-index: the g-index.. ISSI Newsletter.

[6] Kosmulski M (2006). A new Hirsch-type index saves time and works equally well as the original h-index.. ISSI Newsletter.

[7] Jin BH (2006). h-index: an evaluation indicator proposed by scientist.. Science Focus.

[8] Egghe L (2007). Dynamic h-index: The Hirsch index in function of time.. Journal of the American Society for Information Science and Technology.

[9] Liang LM (2006). h-index sequence and h-index matrix: Constructions and applications.. Scientometrics.

[10] Vanclay JK (2006). Refining the h-index.. Scientist.

[11] Iglesias JE, Pecharroman C (2007). Scaling the h-index for different scientific ISI fields.. Scientometrics.

[12] Sidiropoulos A, Katsaros D, Manolopoulos Y (2007). Generalized Hirsch h-index for disclosing latent facts in citation networks.. Scientometrics.

[13] Rousseau R, Ye FY (2008). A proposal for a dynamic h-type index.. Journal of the American Society for Information Science and Technology.

[14] Jin BH, Liang LM, Rousseau R, Egghe L (2007). The R- and AR-indices: Complementing the h-index.. Chinese Science Bulletin.

[15] Bornmann L, Mutz R, Daniel HD (2008). Are there better indices for evaluation purposes than the h index? a comparison of nine different variants of the h index using data from biomedicine.. Journal of the American Society for Information Science and Technology.

[16] Bar-Ilan J (2008). Informetrics at the beginning of the 21st century - A review.. Journal of Informetrics.

[17] Bornmann L, Daniel HD (2009). The state of h index research Is the h index the ideal way to measure research performance?. Embo Reports.

[18] Rousseau R (2006). New developments related to the Hirsch index.. Science Focus.

[19] Peterson A (2007). H-index ranking of living chemists.. Chemistry World.

[20] Egghe L (2008). Mathematical theory of the h- and g-index in case of fractional counting of authorship.. Journal of the American Society for Information Science and Technology.

